# Clinical presentation and engagement with a cognitive rehabilitation clinic in individuals reporting anomalous health incidents

**DOI:** 10.3389/fneur.2026.1858023

**Published:** 2026-08-26

**Authors:** Kelly Marshall, Katherine Sullivan, Louis M. French

**Affiliations:** 1 National Intrepid Center of Excellence, Walter Reed National Military Medical Center, Bethesda, MD, United States; 2 Uniformed Services University of the Health Sciences, Bethesda, MD, United States

**Keywords:** anomalous health incident, clinical outcomes, cognition, cognitive rehabilitation, cognitive training, symptom reporting

## Abstract

**Introduction:**

The Department of Defense is working to establish effective rehabilitative management options for individuals who have experienced Anomalous Health Incidents (AHI).

**Methods:**

This retrospective, observational study conducted at Walter Reed National Military Medical Center describes the clinical presentation of forty patients (21 female and 19 male; mean [SD] age, 43.3 [9.2] years) with reported AHI. It also describes the application of a cognitive training intervention, its utilization, patient satisfaction, and changes in cognitive performance and symptoms over time. At baseline, the overall pattern of objective cognitive efficiency scores was within normal limits, though numerically below healthy norms. However, patients performed below normal limits on measures of processing speed. Patient self-report surveys found slightly elevated psychological symptoms, highly elevated neurobehavioral and headache symptoms, and severe impact on life.

**Results:**

Patients used an at-home cognitive training program and provided generally positive feedback regarding the program. On an internal clinic survey, over 75% of the patients felt that participation helped in areas of memory, visuo-spatial skills, and attention. Some changes in objective cognitive testing were observed over time. Self-reported symptoms, which were not directly targeted in the intervention, did not change over time.

**Discussion:**

This study demonstrates that the clinical integration of targeted cognitive training for individuals reporting cognitive symptoms following an AHI is feasible and acceptable, with patients demonstrating good engagement and a positive clinical trajectory in cognitive efficiency over time.

## Introduction

In 2017, some United States (US) Government employees stationed in Cuba reported unusual sensory experiences, typically accompanied by the sudden onset of symptoms such as vertigo, head pain, tinnitus, or cognitive changes. Initially referred to as “Havana syndrome,” the US government now refers to these events as Anomalous Health Incidents (AHIs). The etiology of these symptoms is unclear, and various explanations have been posited, to include the effects of a directed attack by a malign actor, existing premorbid health or environmental concerns, or psychogenic illness, among other possibilities (1).

Few published studies have examined objective neuropsychological performance and cognitive symptom reporting in this population. A 2018 study of 24 individuals with suspected AHIs reported abnormalities on objective neuropsychological testing, as well as persistent subjective reports of headache, sleep, and cognitive symptoms (2). Another study of 86 participants with reported AHI revealed that, compared to a control group, there were no significant differences on neuropsychological test performance; however, there were higher subjective symptoms of posttraumatic stress, fatigue, and depressive symptoms (1). While these two studies reached different conclusions about the presence of objective neuropsychological findings, both corroborated high rates of symptoms, which alone can have a direct impact on quality of life and return to work for individuals. Indeed, a longer term follow up of some subjects in the former study (3) demonstrated chronic symptom reporting and reduced return to work in some of the US Diplomats who had reported an AHI. Specifically, sensory, cognitive, sleep, and vestibular symptoms were associated with an inability to work. Some never returned to work (15%) and others (22%) were out of work for some period. These functional impacts demonstrate the importance of investigating interventions that address objective performance and/or symptoms in patients reporting AHI.

Federal law requires the Department of Defense (DoD) to provide care to government employees and their families reporting symptoms following an AHI (4). The Defense Health Agency (DHA) has developed clinical recommendations for primary care providers treating individuals who report ongoing cognitive and behavioral symptoms following an incident (5). Evaluation considerations include ordering the computerized neurocognitive assessment, Automated Neuropsychological Assessment Metrics (ANAM) and symptom-report questionnaires, specifically the Patient Health Questionnaire-9 (PHQ-9) and the Generalized Anxiety Disorder-7 (GAD-7). Symptom management considerations include mindfulness, alternative work schedules, referrals to behavioral health, and follow-up ANAM assessments for comparisons. The DoD Clinical Practice Guidelines for mild traumatic brain injury (mTBI) are also recommended for treatment consideration (6).

As of April 2024, 257 individuals had received approvals for care in the DoD military health system (7). This paper describes a subset of those patients and additional patients since 2024 who were referred for cognitive intervention to the Brain Fitness Center (BFC), a service of the National Intrepid Center of Excellence (NICoE) at Walter Reed National Military Medical Center (WRNMMC) in Bethesda, Maryland. The BFC administers assessment measures over time including the recommended ANAM, PHQ-9, and GAD-7. In addition, the BFC offers an intervention modality to train cognitive functioning that is also recommended by the DoD. We describe demographic characteristics, cognitive performance, and symptom reporting of patients referred for cognitive intervention following a reported AHI. We also describe the intervention offered, utilization, and satisfaction. Finally, we examine changes in symptoms over time and changes in cognitive performance during the use of a targeted intervention. The aims of this observational study are to (1) explore the clinical presentation of patients with cognitive concerns following potential exposure to AHI and (2) to describe patient engagement, acceptability, and the clinical trajectory of cognitive symptoms while patients utilized a targeted cognitive training intervention. Results will provide preliminary data that advances the understanding the clinical care needed in this complex population.

## Materials and methods

This is a retrospective, observational study. The protocol (WRNMMC-EDO-2022-0941) was deemed exempt by the Walter Reed Institutional Review Board on September 13, 2022, under Exempt Category 4 (Secondary Research). The exemption was granted because the project constitutes secondary research using an existing clinical data set maintained by National Intrepid Center of Excellence Informatics, where the identity of the human subjects cannot readily be ascertained. Per Walter Reed IRB protocol, a de-identified data set of all patients reporting an AHI who were seen at the BFC was requested for use in this study. Patients received the typical standard of care at the BFC.

### Participants and setting

Forty US government employees and their adult family members referred to the BFC at WRNMMC between December 2020 and May 2025 were included. The current DoD Clinical Algorithm for Management of AHI requires that the individual should have experienced an AHI Sensory Event characterized by the “Sudden experience of pressure, loud sounds, or other auditory phenomena concurrently or immediately preceding the new onset of one or more symptoms, including, but not limited to (Cognitive problems [e.g., attention problems, difficulty concentrating, brain fog, disorientation], Headache and/or head pressure, Nausea, Otologic symptoms [e.g., ear pain, tinnitus, fullness, pressure, hearing loss], Vestibular dysfunction [e.g., dizziness, vertigo, imbalance, illusion of movement], Vision changes [e.g., double vision, blurred vision]; Symptoms may improve upon moving away from where symptoms began and may or may not be experienced by others in the same area, AND Symptoms are not fully explained by other apparent environmental or medical conditions” (5). This study utilized a pragmatic, operational inclusion criterion: individuals treated at the NICoE who were referred specifically for symptoms related to a potential or suspected AHI exposure. However, because there are no universally accepted diagnostic criteria or validated biomarkers for AHI, and these patients originated from various federal departments and agencies, the initial identification and classification of a potential AHI were conducted upstream by their parent agencies or primary care managers based on varying internal protocols. Consequently, patients did not undergo a singular, standardized diagnostic process prior to referral, and no specific exclusionary criteria regarding AHI etiology were applied by the BFC.

Demographic characteristics of the patients are presented in Table 1. All patients referred had expressed cognitive concerns and were seeking intervention. The BFC is a supplemental cognitive rehabilitation clinic that is a resource for patients with subjective cognitive concerns, with or without a diagnosis of a neurological injury, disease, or disorder (8). Patients may also be receiving other types of medical or rehabilitative care, according to their individual treatment plans.

**Table 1:** Patient demographic information.

Demographic characteristics		
Characteristic (*n* = 40)	Avg.	SD
Age	43.3	9.2
	*n*	%
Sex		
Female	21	52.5
Male	19	47.5
Military status		
Active Duty	5	12.5
Retired	5	12.5
Dependent	1	2.5
Non-military	29	72.5
Marital status		
Single	9	22.5
Married	26	65
Divorced	5	12.5
Education level		
High School	3	7.5
Some College	2	5
College	12	30
Master’s Degree	15	37.5
Advanced	7	17.5
Unknown	1	2.5
Time post incident (months)		
≤6	6	15
7 to 12	6	15
13 to 24	13	32.5
≥25	15	37.5

**Figure 1:**
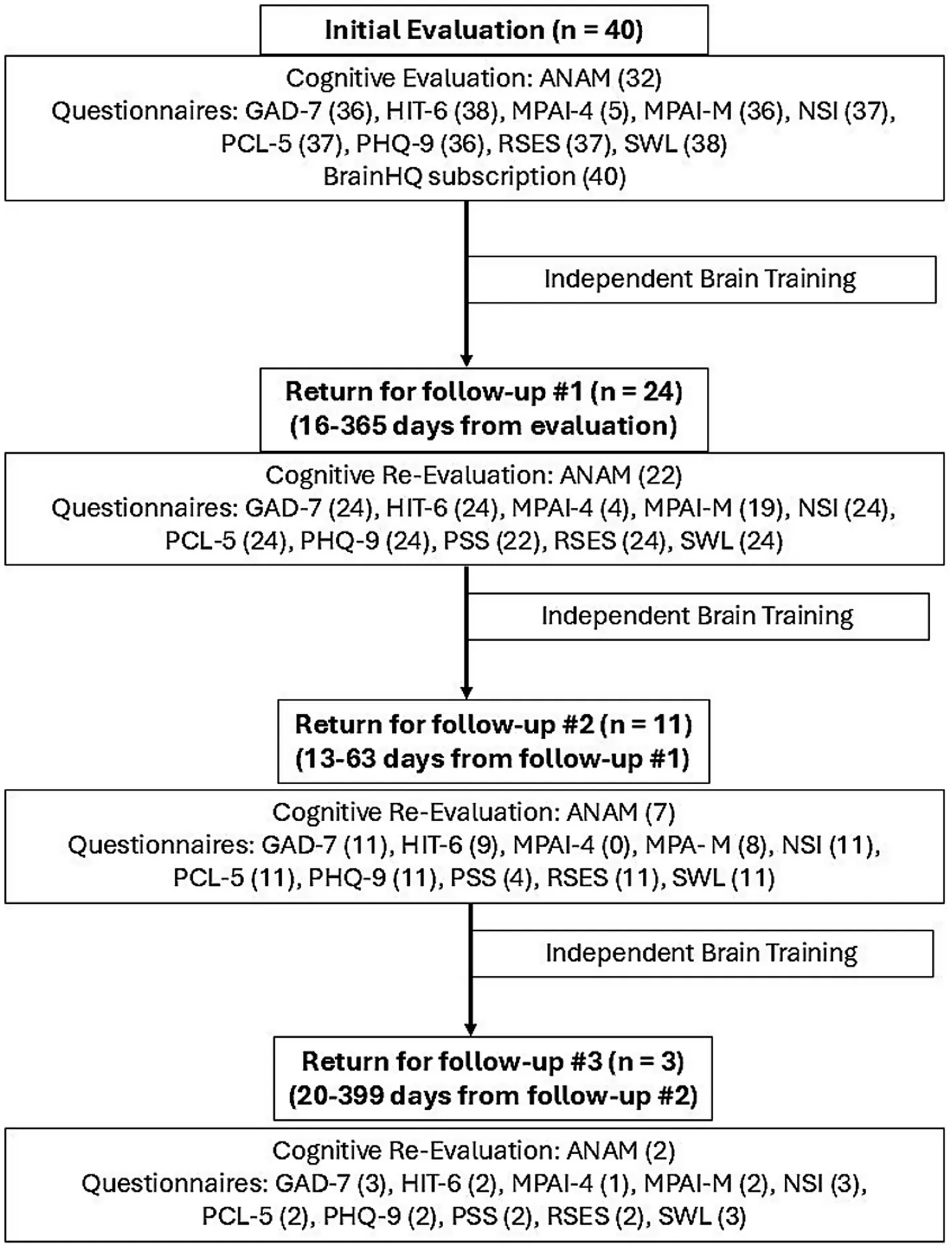
Participation in the Brain Fitness Center. Objective and subjective assessment measures are shown for each visit with the number of patients completing that measure included in parentheses. ANAM, Automated Neuropsychological Assessment Metrics; GAD-7, Generalized Anxiety Disorder 7-Item Scale; HIT-6, Headache Inventory Test; MPAI4, The Mayo-Portland Adaptability Inventory-4; MPAIM, The Mayo-Portland Adaptability Inventory-M; NSI, Neurobehavioral Symptom Inventory; PCL-5, Post-Traumatic Stress Disorder (PTSD) Checklist, DSM-5; PCL-C, Post-Traumatic Stress Disorder (PTSD) Checklist, Civilian Version; PHQ-9, Patient Health Questionnaire-9; PSS, Patient Satisfaction Survey; RSES, Responses to Stressful Experiences Scale; SWLS, Satisfaction with Life Survey.

Patients referred to the BFC complete a clinical interview, a standard cognitive assessment (ANAM), and a series of symptom-self report surveys. Demographic information is collected by patient interview and medical chart review. The administration of the cognitive assessment is completed on an ANAM-specific laptop (selected by the military for standardized processing and timing) and supervised by a BFC staff member. All symptom surveys are completed on a desktop computer in the BFC. Note that not all symptom surveys are used as direct measures for cognitive intervention but are administered to monitor changes in patients’ mental or physical health. Following the interview and assessments, a clinician meets with each patient and provides individual recommendations for using a computer-based cognitive training tool. The cognitive training programs can be used in the BFC or at home. Follow-up assessments are scheduled for those that live close to the WRNMMC or return for other medical appointments. The number of patients participating in each intake and follow-up visit, the time between visits, and the structure of the cognitive training tool use is shown in Figure 1.

### Measures

The ANAM4 Military (ANAM4 MIL-TBI) is the DoD’s neurocognitive assessment of record and the recommended measure to use in the AHI population (5). The Standard ANAM4 Military (“ANAM”) is a computerized battery of seven standard subtests: Simple Reaction Time (SRT; reaction time), Code Substitution (CDS; attention, working memory), Procedural Reaction Time (PRO; reaction time, attention), Mathematical Processing (MTH; attention, working memory), Matching to Sample (M2S; visuospatial working memory), Code Substitution – Delayed (CDD; delayed memory), and SRT (repeated, SR2; reaction time, fatigue). The present study used throughput measures from each subtest and the ANAM Composite Score (ACS) to measure objective cognitive performance. The ACS is based on throughput scores for each subscale and is mathematically similar to an overall test battery mean. The ACS score is a calculated z score adjusted for age and sex (9). Lower scores indicate worse performance. The ACS is considered a good endpoint option in research to quantify global cognitive impairment (10). The throughput score is calculated as a ratio between the number of correct responses per unit time available for the task. The ANAM includes a Performance Validity Index (PVI) to assess the patient’s motivation and effort to perform as well as possible on the test, or understanding of the instructions (11).

The Neurobehavioral Symptom Inventory (NSI) consists of 22 symptom questions commonly reported after concussion (12). Patients are asked to rate symptoms regarding how much they have been disturbed by them in the last 2 weeks on a five-point scale– ranging from 0 (none) to 4 (very severe) for a total range of 0 to 27. The 22-item total score and four factors were used in the analysis: affective (range 0 to 24), cognitive (range 0 to 16), somatic (range 0 to 28), and vestibular (range 0 to 12) (13, 14).

The Headache Inventory Test (HIT-6) is a six-item questionnaire. Patients are asked to rate the severity, frequency, and impact of headaches on a scale of 6 (never) to 13 (always) with a total range of 36 to 78 (15).

The Generalized Anxiety Disorder 7-item (GAD-7) scale is a seven-item self-report anxiety questionnaire that assesses the patient’s anxiety symptoms over the last 2 weeks (16). Patients are asked to rate the symptoms based on how often they are bothered by the problems from 0 (not at all) to 3 (nearly every day) with a total range of 0 to 21.

The Patient Health Questionnaire-9 (PHQ-9) is a 9-item self-administered depression survey. Patients are asked to rate symptoms from 0 (not at all) to 3 (nearly every day) with a total range of 0 to 27 (17).

The Post-Traumatic Stress Disorder (PTSD) Checklist, DSM-5 (PCL-5) is a questionnaire consisting of 20 possible symptoms of PTSD. Patients are asked to rate the symptoms on how bothered they are on a 5-point scale ranging from 0 (not at all) to 4 (extremely) with a total range of 0 to 80 (18).

The Mayo-Portland Adaptability Inventory-M (MPAI-M) is a military-specific questionnaire consisting of 21 areas of abilities, adjustments, and participation in tasks of daily life. Patients are asked to rate the items on a 5-point scale ranging from 0 (none) to 4 (severe problem) with a total range of 0 to 84 (19). Eight patients completed the MPAI-4, which includes all the items on the MPAI-M plus additional questions not part of the MPAI-M. Scores for items that occur on the MPAI-M were used to calculate an MPAI-M score for these patients.

The Response to Stressful Experience Scale (RSES) is a 22-item questionnaire that assesses coping and resiliency. Patients are asked to rate a response to a stressful event ranging from 4 (exactly like me) to 0 (not at all like me) with a total range of 0 to 88 (20).

Satisfaction with Life Survey (SWLS) is a survey that consists of five questions regarding how satisfied patients are with life (21). Patients are asked to rate their agreement with each item using a 7-point scale from 1 (Strongly Disagree) to 7 (Strongly Agree) with a total range of 0 to 84.

### Cognitive training tool

Each patient was offered a subscription to the online cognitive training program, BrainHQ. BrainHQ is neuroplasticity-based training tool that targets adaptations in cognitive domains such as processing speed, working memory, and attention (22). Twenty-nine game-like exercises organized into six cognitive domains (Attention, Brain Speed, Memory, People Skills, Intelligence, and Navigation) provide repetitive and adaptative stimulation to continuously challenge the user. There are currently no studies investigating the use of neuroplasticity cognitive training programs in the AHI population; however, there are published studies in healthy adults (23), and healthy aging adults (24). In addition, there are studies in other patient populations with similar symptom profiles such as traumatic brain injury (TBI) (25, 26), tinnitus (27), attention deficit hyperactivity disorder (ADHD) (28), and depression (29, 30). Clinician-directed computer-based cognitive training within an integrated cognitive rehabilitation treatment plan is recommended by the DoD for populations with similar presentation, such as individuals with mTBI (6). The BFC provided a subscription to BrainHQ to each patient with a general recommendation to use the program for 20 to 30 min, three to five times a week. A clinician met with each patient to review the program and provide recommendations on use related to the patient’s cognitive complaint. The program can be accessed through a URL or on a smartphone application. An administration portal allows clinicians to access patient use and performance.

### Participation satisfaction self-report

The Brain Fitness Center Patient Satisfaction Survey is an internal questionnaire with no associated validity measure. Patients in the BFC are asked if the computer program helped their recovery, if the time commitment was realistic, if the program was fun and engaging, and if it was appropriately challenging. It also asks if they would like the program for home use, if they were glad they participated, and if they would recommend the program. Last, it asks if access to education materials and a brain injury specialist was helpful. Patients are asked these eight questions on a scale from 0 (strongly disagree) to 4 (strongly agree) for a total range of 0 to 28. In addition, patients are asked if the program helped them (yes or no) in the following cognitive domains: Memory, Concentration/Attention, Math, Decision Making, Real-World Tasks, Listening, Visual Tasks, and Vocabulary.

### Statistical analyses

For all analyses involving the ANAM, we excluded data from any ANAM test administration where the results were flagged as potentially invalid (*N* = 3 test sessions). We used an ANAM Performance Validity Index (ANAM-PVI) cutoff of ≥10, in accordance with standards for outpatient samples with cognitive complaints (11). No scores were excluded from subjective symptom report measures. We computed descriptive statistics for the ANAM composite score, each ANAM subtest, and each self-report measure at each available time of administration at the BFC.

Because there is no control group for this retrospective analysis, several analysis methods were used to contextualize the results. For objective measures, we tested whether the average score at each administration of the ANAM (administration 1–4) was significantly different from the normative mean for a non-brain injured active duty military sample (31). For the composite scores, which are z-scores compared to age and sex matched samples stored in the ANAM software, we conducted one-sample t-tests comparing the average composite score to 0. For subtest scores, which are throughput values that are not converted to *z*-scores by the ANAM software, we conducted Welch’s two-sample t-tests comparing the average composite score from our sample to a sample generated from a normative distribution with the same sample size, mean, and standard deviation as reported in Vincent et al. (31). To generate the normative sample values, we used the rnorm function in R. To correct for multiple comparisons, we used a Bonferroni-corrected value of alpha = 0.0016 to account for 32 comparisons over a familywise error rate of 0.05. To calculate effect sizes for each *t*-test, we used the cohens_d function in R (32).

To compare the current sample of patients with an additional cohort of neurologically healthy individuals that may be better matched in terms of education level, we examined patients’ performance relative to published ANAM performance norms for practicing military National Guard and Reserves physicians. Following the convention of Meyers et al. (33), we calculated the proportion of patients who scored “Below Average” or worse (>1.3 standard deviations) on two or more subtests in order to examine whether the proportion of patients with abnormal scores was higher than the base rate of abnormal test results in a healthy population.

For the subjective self-report measures, published reference values are primarily provided to describe how score ranges correspond to symptom severity. Other measures provide symptom severity cutoffs. Given that this information does not allow the same kinds of analyses as completed for the objective measures, we instead describe the score distributions relative to available published values for each measure; for the RSES scale, we used internal NICoE clinic conventions for score ranges (13, 15–17, 19–21, 34).

To assess the stability of objective assessments and subjective symptom report over repeated visits to the BFC, we used the lme4 package in R to construct a linear mixed effects model assessing the effect of measure administration number on each measure with a random intercept of patient. We could not use the maximal random effects structure because models would not converge with random slopes of test number by participants due to missing data (i.e., different participants have different numbers of test administrations). Administration 1 was set as the reference level. We used the emmeans package in R to complete Tukey’s HSD pairwise comparisons (35, 36).

There are several considerations to note for the approach to modeling the change in clinical presentation. First, individual patients may vary in a variety of ways that could affect objective or subjective scores and the clinical trajectory of these scores. Given the size and complexity of this dataset, we captured individual variability using random effects of patients (37). The available dataset could not support a more complex model including interactions between covariates and administration or modeling time-varying factors like post-traumatic stress symptoms. We also did not include specific covariates to avoid overfitting the models to the current dataset, which may not be representative of all individuals with AHI. Second, we report the results for each administration of each measure to reflect general changes over repeated interactions with rehabilitation services. We use this framework rather than changes over time, given the high variability in time between visits across patients. Using administration rather than time means that the amount of time between each administration varied by patient. If a patient did not complete a measure during a particular visit to the BFC, this means that the administration number for a measure at a given session may not match the administration numbers for other measures at the same session (e.g., a patient who did not complete the ANAM but did complete the PCL-5 on visit 1 and completed both on visit 2 would have ANAM number 1 and PCL-5 number 2 occur at the same visit time).

### Cognitive training tool utilization

We extracted information about BrainHQ use for the subset of patients whose information was available in the online administrative portal. We calculated the number of days of use and average time per day for each patient, which we then used to calculate descriptive statistics for the group.

## Results

### Clinical profile

#### Objective cognitive performance

**Figure 2:**
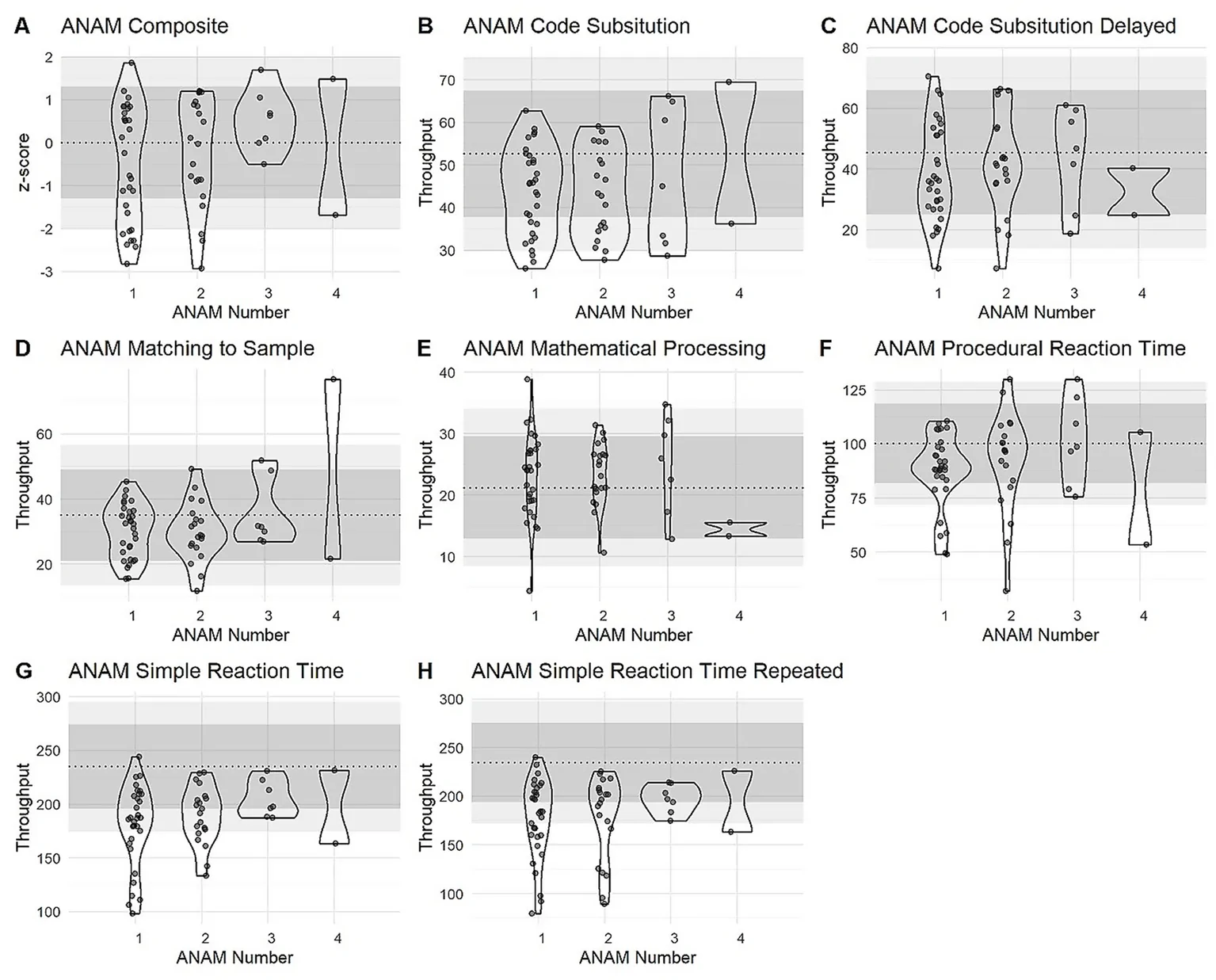
Distribution of scores on repeated administrations of the ANAM. ANAM number represents the sequential administration of the ANAM (e.g., ANAM number 2 is the second time a patient took the ANAM). **(A)** The ANAM composite score is a weighted average of the ANAM subtest scores, *z*-scored relative to age- and sex-matched normative sample. **(B–H)** Throughput scores represent correct responses per minute of available response time. Outlines show the distribution of the data. Points represent individual patients’ scores. Dark grey boxes represent +/− 1.3 standard deviations from the mean normative value. Light grey boxes represent +/− 2 standard deviations from the mean normative value (31). Scores within the dark grey box represent an “average or above” score, scores below the dark grey box but within the light grey box represent a “below average” score, and scores below either grey box represent a “clearly below average” score, as would be indicated on an ANAM report.

The majority of patients’ scores (>50%) on the ANAM composite and subtest throughput scores on Code Substitution, Code Substitution Delayed, Matching to Sample, Mathematical Processing, and Procedural Reaction Time fell within the range expected based on neurologically healthy norms (31) (Figure 2). This reflects generally intact performance for tests of attention, working memory, and delayed memory. Although most individual scores would be considered within normal limits, on average, patients did perform significantly worse than the normative sample on the code substitution (1st and 2nd administration) and procedural reaction time (1st administration) throughput measures with large effect sizes (Table 2).

**Table 2:** Summary of results of one-sample *t*-tests to determine whether group performance on objective cognitive measures differed from the mean performance of the normative sample (31).

Subtest	ANAM Number	Sample Mean	Normative Mean	Statistic (*t*)	df	*p*-value	Cohen’s d
ANAM composite	1	−0.49	0	−1.99	30	0.06	−0.36
	2	−0.26	0	−0.92	20	0.37	−0.20
	3	0.52	0	1.88	6	0.11	0.71
	4	−0.11	0	−0.07	1	0.10	−0.05
ANAM code substitution	1	43.63	52.6	−4.77	30.02	**<0.001**	−0.78
	2	43.37	52.6	−4.00	19.01	**<0.001**	−0.81
	3	47.14	52.6	−0.88	6.00	0.41	−0.48
	4	52.85	52.6	0.02	1.00	0.99	0.02
ANAM code substitution delayed	1	38.29	45.4	−2.50	30.02	0.02	−0.45
	2	41.76	45.4	−0.97	19.01	0.34	−0.23
	3	43.91	45.4	−0.23	6.00	0.82	−0.09
	4	32.51	45.4	−1.66	1.00	0.34	−0.81
ANAM matching to sample	1	30.07	35.0	−3.31	30.03	0.002	−0.46
	2	29.96	35.0	−2.50	19.01	0.02	−0.47
	3	35.36	35.0	0.07	6.00	0.94	0.04
	4	49.14	35.0	0.51	1.00	0.70	1.31
ANAM mathematical processing	1	22.95	21.2	1.45	30.02	0.15	0.27
	2	23.39	21.2	1.95	19.01	0.07	0.35
	3	24.99	21.2	1.26	6.00	0.25	0.60
	4	14.4	21.2	−6.25	1.00	0.10	−1.06
ANAM procedural reaction time	1	87.49	100.2	−4.19	30.01	**<0.001**	−0.90
	2	92.02	100.2	−1.58	19.00	0.13	−0.58
	3	101.46	100.2	0.16	6.00	0.88	0.09
	4	79.38	100.2	−0.80	1.00	0.57	−1.47
ANAM simple reaction time	1	179.91	234.8	−8.07	30.01	**<0.001**	−1.82
	2	189.61	234.8	−7.53	19.01	**<0.001**	−1.50
	3	204.98	234.8	−4.54	6.00	0.003	−0.98
	4	197.14	234.8	−1.10	1.00	0.47	−1.25
ANAM simple reaction time repeated	1	176.70	234.6	−7.86	30.01	**<0.001**	−1.86
	2	177.70	234.6	−5.83	19.00	**<0.001**	−1.82
	3	197.00	234.6	−6.75	6.00	**<0.001**	−1.21
	4	194.67	234.6	−1.27	1.00	0.42	−1.28

Using Bonferroni correction, we used an alpha of 0.0016 to determine significance, given a familywise error rate of 0.05 and 32 comparisons. *p*-values are shown in bold when they were below the corrected significance threshold.

However, more than 50% of scores on the Simple Reaction Time and Repeated Simple Reaction Time would be classified as “below average” or “clearly below average” according to ANAM scoring conventions (>1.3 standard deviations less than the mean; Figure 2). On average, patients performed significantly worse than the normative sample on these tasks (Simple Reaction Time: 1st, 2nd administration; Simple Reaction Time Repeated: 1st, 2nd, 3rd administration), with large effect sizes (Table 2).

When examining individuals’ performances, there was a high proportion of patients who scored “Below Average” or worse on two or more subtests on any given administration (minimum 43%, maximum 54%). This is a notably higher proportion than that reported in the ANAM physician norms (11.2%) (33).

#### Subjective self-report

**Figure 3:**
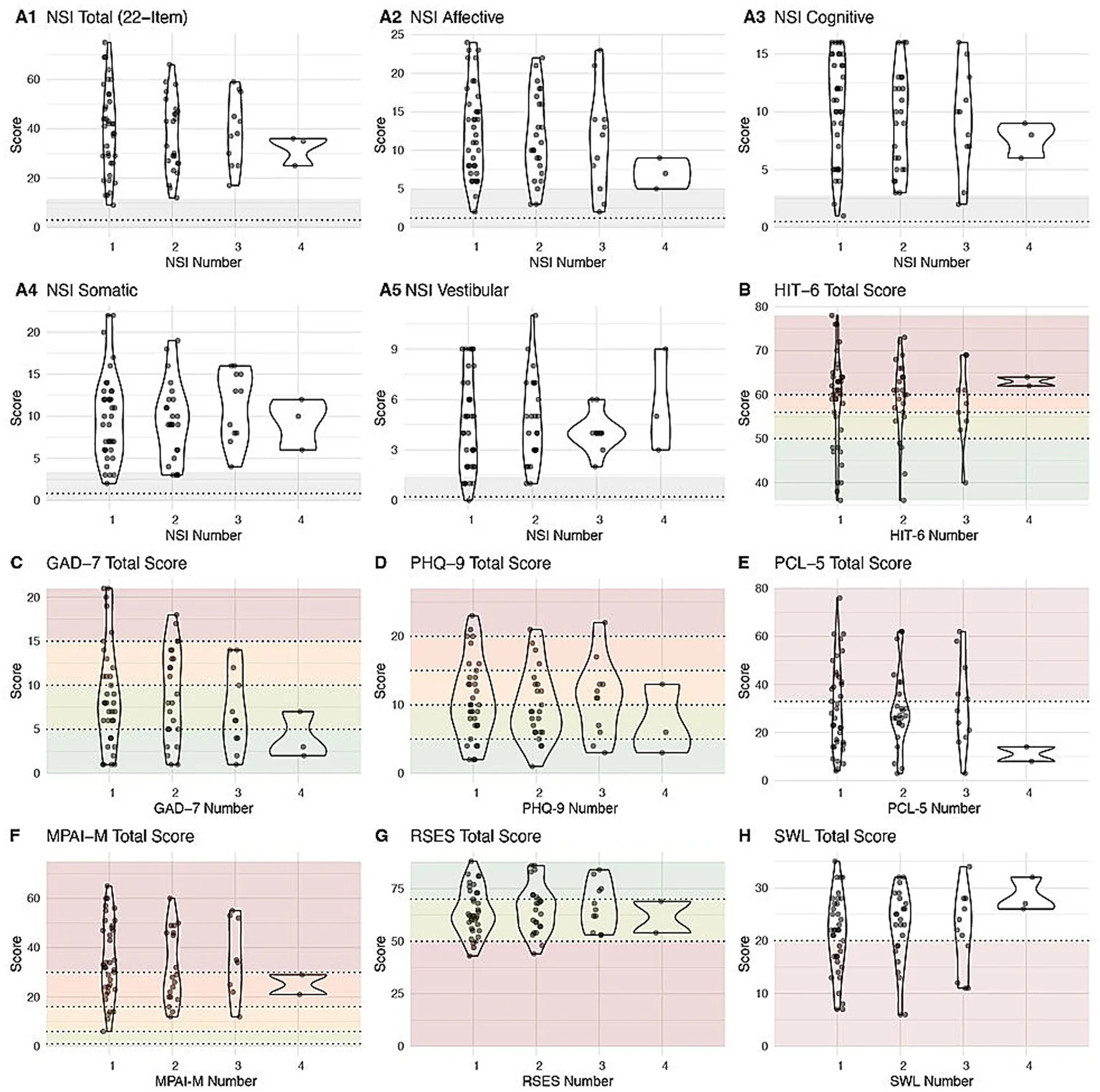
Distribution of scores on repeated administrations of each symptom self-report measure. For each panel, measure number represents the sequential administration of the measure (e.g., for PCL-5, PCL-5 number 2 is the second time a patient took the PCL-5). Outlines show the distribution of the data. Points represent individual patients’ scores. NSI. Panel **A1** shows the NSI total score, including all 22 items. Panels **A2–A5** show subscale factor scores (14). Dotted horizontal lines represent the mean normative value for the non-clinical, non-deployed National Guard sample. Grey boxes represent +/− 1.5 standard deviations from the mean normative value (13). **(B)** HIT-6. Dotted horizontal lines represent cutoff points for severity interpretations. Dark green shading represents “little to no impact,” light green shading represents “moderate impact,” orange shading represents “substantial impact,” red shading represents “severe impact” (15). **(C)** GAD-7. Dotted horizontal lines represent cutoff points for severity interpretations. Dark green shading represents “minimal anxiety,” light green shading represents “mild anxiety,” orange shading represents “moderate anxiety,” red shading represents “severe anxiety” (16). **(D)** PHQ-9. Dotted horizontal lines represent cutoff points for severity interpretations. Dark green shading represents “minimal depression,” light green shading represents “mild depression,” yellow shading represents “moderate depression,” orange shading represents “moderate to severe depression,” red shading represents “severe depression” (17). **(E)** PCL-5. Dotted horizontal line represents the suggested cutoff for possible PTSD in primary care clinics (34, 38). Red shading represents scores above the cut point. **(F)** MPAI-M. Dotted horizontal lines represent cutoff points for severity interpretations. Dark green shading represents “good participation,” light green shading represents “mild limitations to participation,” yellow shading represents “mild to moderate limitations to participation,” orange shading represents “moderate to severe limitations to participation,” red shading represents “severe limitations to participation” (19). **(G)** RSES. Dotted horizontal lines represent cutoff points for interpretations. Green shading represents “high resilience,” yellow shading represents “moderate resilience,” red shading represents “low resilience” ((20); NICoE scoring conventions). **(H)** SWL. Dotted horizontal lines represent the cutoff score for neutral satisfaction with life (21). Red shading represents scores with worse than neutral satisfaction with life.

*Neurobehavioral symptom inventory (NSI)*. Across all administrations of the NSI, patients’ total scores and scores on each of the subtests were substantially higher than normative scores from a comparison sample of non-clinical, non-deployed National Guard members (13). Between 75–100% of scores were more than 1.5 standard deviations from the normative mean across all administrations of the NSI, indicating substantial symptom severity across affective, cognitive, somatic, and vestibular domains (Figures 3A1–A5; Table 3).

**Table 3:** Descriptive statistics for objective and subjective measures.

Measure	Administration	*N*	Mean	Standard deviation
ANAM composite (*z* score)	1	31	−0.49	1.37
	2	21	−0.26	1.28
	3	7	0.52	0.73
	4	2	−0.11	2.24
ANAM code substitution (Throughput)	1	31	43.63	10.39
	2	21	43.37	10.31
	3	7	47.14	16.50
	4	2	52.85	23.55
ANAM Code substitution delayed (Throughput)	1	31	38.29	15.85
	2	21	41.76	16.66
	3	7	43.91	16.76
	4	2	32.51	10.96
ANAM match to sample (Throughput)	1	31	30.07	8.28
	2	21	29.96	9.03
	3	7	35.36	10.32
	4	2	49.14	38.98
ANAM mathematical processing (Throughput)	1	31	22.95	6.70
	2	21	23.39	5.07
	3	7	24.99	7.97
	4	2	14.4	1.54
ANAM procedural reaction time (Throughput)	1	31	87.49	16.86
	2	21	92.02	23.09
	3	7	101.46	20.37
	4	2	79.38	36.88
ANAM simple reaction time (Throughput)	1	31	179.91	37.84
	2	21	189.61	26.83
	3	7	204.98	17.28
	4	2	197.14	48.15
ANAM simple reaction time repeated (Throughput)	1	31	176.70	41.02
	2	21	177.70	43.56
	3	7	197.00	14.72
	4	2	194.67	44.19
GAD-7	1	36	8.83	5.78
	2	24	9.04	5.25
	3	11	7.27	4.61
	4	3	4.00	2.65
HIT-6	1	38	58.55	10.47
	2	24	59.25	8.88
	3	9	57.78	8.94
	4	2	63.00	1.41
MPAI-M	1	36	35.19	15.91
	2	19	31.95	14.73
	3	8	36.00	16.05
	4	2	25.00	5.65
NSI total	1	37	39.19	17.51
	2	24	37.38	15.09
	3	11	39.09	14.00
	4	3	32.00	6.08
NSI affective	1	37	12.49	5.95
	2	24	11.75	5.57
	3	11	11.27	6.78
	4	3	7.0	2.00
NSI cognitive	1	37	10.22	4.49
	2	24	9.17	4.27
	3	11	9.27	4.47
	4	3	7.67	1.53
NSI somatic	1	37	9.94	5.17
	2	24	9.33	4.66
	3	11	11.27	4.19
	4	3	9.33	3.06
NSI vestibular	1	37	4.38	2.77
	2	24	4.75	2.59
	3	11	4.09	1.14
	4	3	5.67	3.06
PCL-5	1	37	30.68	18.26
	2	24	29.79	15.92
	3	11	31.64	18.18
	4	2	11.00	4.24
PHQ-9	1	36	10.76	5.71
	2	24	9.83	5.13
	3	11	10.82	5.65
	4	3	7.33	5.13
RSES	1	37	64.20	11.04
	2	24	65.46	11.64
	3	11	66.55	11.14
	4	2	61.50	10.60
SWL	1	38	21.19	7.26
	2	24	22.29	7.29
	3	11	21.45	7.65
	4	3	28.33	3.21

*Headache Impact Test-6 (HIT-6).* Patients’ reports of their headache severity reflected scores that fell primarily in the severe impact range (15) (Figure 3B; Table 3).

*Generalized Anxiety Disorder 7-item (GAD-7)*. Most patients’ scores (>50%) fell within the minimal to mild anxiety range (16). (Figure 3C; Table 3).

*Patient Health Questionnaire-9 (PHQ-9)*. Across administrations of the PHQ-9, the majority of patients’ scores fell within the minimal to mild depression range (17) (Figure 3D; Table 3).

*Post-traumatic stress disorder checklist, DSM-5 (PCL-5)*. The majority of patients (>50%) did not meet the cutoff for likely PTSD, although the mean score for administration 1 (30.68) was close to the cut score of 31 indicating “probable” PTSD (34, 38) (Figure 3E; Table 3).

*Mayo-portland adaptability inventory, military (MPAI-M)*. Across administrations of the MPAI-M, more than 50% of patients indicated that their symptoms led to moderate-to-severe or severe limitations in their daily functioning (19) (Figure 3F; Table 3).

*Responses to stressful experiences scale*. More than 75% of patients’ scores fell within the moderate or high resilience ranges on all administrations of the RSES (20) (Figure 3G; Table 3).

*Satisfaction with life scale*. Across administrations of the SWL, most patients (>50%) reported having better than neutral satisfaction with life (21) (Figure 3H; Table 3).

### Clinic participation and engagement in the intervention

Due to time, tele-health visits, or other factors, not all patients completed all assessments. Twenty-four of the patients who attended an initial intake appointment (60% of original sample) returned for one follow-up evaluation. The 16 who did not return either did not feel they needed additional intervention or were no longer located in the area. Of those 24, 11 returned for a second follow-up evaluation (28% of original sample), and of those 11, three returned for a third follow-up evaluation (8% of original sample). Figure 1 shows the clinical activities completed during each visit and number of patients participating.

The BrainHQ program can be monitored remotely using an administrative portal. Utilization data was available for 25 individuals. The remaining 15 patients either never used the program or changed their account information, and we were therefore unable to access their data. All patients with available data used the tool at least once. Patients used the program for approximately 1.5 months total (Table 4). Patients typically engaged with BrainHQ for less than 30 min per session, which is in line with recommendations provided by BFC staff when demonstrating the tool. These results demonstrate engagement with the cognitive tool provided and general compliance with BFC recommendations within the AHI population.

**Table 4:** Descriptive statistics for cognitive training tool use for patients with available data.

	*N*	Mean	SD	Min	Max
Days used	25	48.60	86.97	1	399
Minutes/day	25	21.73	19.43	1.72	99.62

### Change in performance and self-report

There was a significant change in objective cognitive scores on follow-up administrations of the ANAM for the composite score (1 vs. 3: b = −1.03, *p* = 0.014) and the following subscale scores: Code Substitution (1 vs. 4: b = −9.15, *p* = 0.027), Matching to Sample (1 vs. 4: b = −18.06, *p* = 0.001; 2 vs. 4: b = −17.72, *p* = 0.012), and Procedural Reaction Time (1 vs. 3: b = −16.97, *p* = 0.042). The direction of change reflects a positive clinical trajectory. There was no significant change in scores for the Code Substitution Delayed, Mathematical Processing, Simple Reaction Time, or Simple Reaction Time Repeated subscales (Figure 2; Table 3).

There were no significant changes in any of the self-report scores across administrations (Figure 3; Table 3).

### Participation satisfaction self-report

Patient Satisfaction was examined for the average rating across all eight questions and for each question individually. We use the PSS form from the first follow-up evaluation. Overall, patients were satisfied (total mean = 3.07 out of maximum 4, standard deviation [SD] = 0.39). The average response ratings per question are shown in Table 5. The satisfaction with individual questions was varied, with a satisfaction range (2.5–3.7) compared to the overall total mean of 3.07.

**Table 5:** Summary of responses to the Patient Satisfaction Survey (PSS) at first follow-up.

Patient satisfaction survey statement	*n = 22*	
	Overall mean = (3.08)	SD = (0.39)
	Mean	SD
This computer program helped my recovery process.	2.82	0.66
I thought the time commitment to the program was realistic and easy to accomplish.	2.55	1.01
I found the content of the computer program to be fun and engaging.	2.73	0.88
I found the content of the computer program to be appropriately challenging	3.18	0.85
I would like to have this computer program at home.	2.91	0.97
I am glad I participated in this program.	3.32	0.71
I would recommend this computer program to other service members.	3.45	0.67
Access to education materials and brain injury specialists was helpful.	3.68	0.65

0 = Strongly disagree; 1 = disagree; 2 = undecided; 3 = agree; 4 = strongly agree.

In response to the set of yes/no questions, the majority of patients reported that the program was helpful with improving memory (82.6%), visual tasks (82.6%), and concentration/attention (78%). Fewer patients found the program to be beneficial in decision making (43.4%), math skills (4.3%), and vocabulary (13%).

## Discussion

This study describes a sample of individuals being treated clinically following their report of an anomalous health incident and who presented with subjective report of cognitive dysfunction. This study aims to (1) add to the existing literature in establishing a clinical profile for this population and (2) provide preliminary evidence about the clinical feasibility and acceptability of an intervention modality recommended by the DoD and the clinical trajectory of objective and subjective cognitive symptoms during participation with this modality.

### Clinical profile

The sample in our study is diverse, as there exist no accepted diagnostic criteria for this syndrome, individuals were referred at different time points post incident, and varied in age, background, and education. Additionally, individuals were referred for care from multiple Federal departments and agencies, all of whom may have had different criteria in screening for AHI and for referral. However, this referral-based operational definition accurately reflects the real-world clinical landscape. For clinical rehabilitation providers in both military and civilian settings, the specific etiology of a suspected AHI may remain unknown. Therefore, the clinical imperative must be to address the patient’s presenting symptoms and measurable objective performance deficits, rather than definitive diagnostic biomarkers.

There are currently two published reports that include objective data about cognitive performance in this population. In the first (2), of 21 individuals treated, 17 reported cognitive concerns. Of those patients, six had neuropsychological testing with scores reported. It was reported that all “had significant areas of cognitive weakness and/or impairment.” However, a letter in response to the paper (39) noted the data did not appear to support that conclusion, as a non-standard definition of impairment was used. In the second study (1), research participants also reported high levels of cognitive concerns but did not appear significantly different than a control group. In the current analysis of a different sample and using a different cognitive evaluation battery, we also found that the majority of patients’ scores on the ANAM composite and subtest throughput scores fell within the clinically expected range, relative to a normative sample (31). However, patients did perform significantly worse, numerically, than the normative sample on the code substitution (1st and 2nd administration) and procedural reaction time (1st administration) throughput measures with large effect sizes. Additionally, more than 50% of scores on the simple reaction time and repeated simple reaction time would be classified as “below average” or “clearly below average” according to ANAM clinical scoring conventions (>1.3 standard deviations from the mean). In comparison to a different normative comparison group of highly educated individuals (33), there was a higher proportion of patients in the current study who scored “Below Average” or worse on two or more subtests on any given administration.

The reasons for the differences in cognitive functioning noted here on some of the subtests is likely due to several factors. The ANAM is briefer than a full neuropsychological battery, with conclusions about difficulties in a domain predicated on a single test measure, as opposed to a composite comprising several tests across a more comprehensive battery. Also, emotional distress plays a significant role in cognitive functioning and may account for some of the scores noted here (40–42). Of interest, the area where the greatest number of individuals performed below average was on simple reaction time. Simple reaction time is related to how quickly a person can respond to a basic stimulus. As more complex measures of attention and memory (including procedural reaction time) were largely intact, the difficulties noted here may be related to fatigue, alertness, or in this circumstance a reflection of some of the symptoms that patients reported as being elevated, such as headache. It may also be the case that relative differences in processing speed are more apparent in tasks with higher expected throughputs than in more complicated tasks with lower expected throughputs. A more comprehensive battery with composite scores may better account for processing speed across task demands.

As in the previous reports, patients reported elevated levels of distress. Despite generally high resilience, patients reported high levels of cognitive symptom severity and a large impact on life participation. Of note, the level of symptom severity in several domains exceeds that noted in the NIH sample (1). For example, posttraumatic stress symptoms appear more prominent. Elevated posttraumatic stress symptoms, even when they do not reach the level of a full diagnosis, have significant clinical implications (43). Overall neurobehavioral symptoms, as measured by the NSI, are also higher in this group than in previous reports. These high rates of symptoms can have a direct impact on quality of life and return to work in this population (3).

### Response to the intervention modality

Positive clinical trajectories in some areas of objective cognitive performance, as measured by the ANAM, were noted following intervention. There was a significant change for the overall composite score and some subscale scores: Code Substitution, Matching to Sample, and Procedural Reaction Time. These changes were observed while patients participated in an intervention that trained speed and accuracy of information processing. While we cannot draw firm conclusions about the causal relationship between the cognitive intervention and change in objective scores over time (see Limitations section), these results are noteworthy in that they suggest that improvements in objective cognitive performance are possible for patients receiving healthcare interventions after reporting an AHI.

While these improvements were observed alongside participation in the cognitive training intervention, they must be interpreted with caution. First, improvements in objective performance could also be attributed to other concurrent clinical interventions or natural recovery over time since the incident. Second, repeated administrations of computerized neurocognitive assessments can introduce practice effects, wherein improvements reflect increased test familiarity. However, Vincent et al. (44) demonstrated that while small practice effects on the ANAM are expected, a change greater than 0.9 standard deviations on the ANAM Composite Score likely represents a meaningful clinical change. In our sample, the estimated improvement on the ANAM Composite Score between the first and third administration was approximately 1.03 standard deviations (b = −1.03), suggesting that the observed improvements may reflect meaningful clinical change beyond mere practice effects.

Subjective cognitive symptoms did not change over repeated administrations. The training program utilized in this study does not target patients’ self-appraisal of functioning nor symptoms addressed in many of the self-report measures (e.g., traumatic stress, depression, headaches, anxiety). Improvements in such symptoms or daily function may occur based on time from injury or other interventions but are not expected directly from a cognitive training program. Concurrent interventions are likely needed to address possible negative metacognitive bias (45) and factors that impact cognitive function such as stress, sleep, and pain. While unchanged over time, high NSI and PCL-5 scores, coupled with generally low depression and anxiety scores, and high satisfaction with life and resiliency scores are noteworthy clinical features.

Based on internal clinical feedback, the cognitive intervention appeared acceptable to the patient group, with the majority of patients reporting that they felt the clinician-directed computer training program was helpful in improving their memory, visual skills, and concentration/attention, with less efficacy in decision making, math skills, and vocabulary—three domains not overtly addressed in the program. This suggests a generally positive reception to the program and participation in care at the BFC, though these self-reported impressions must be interpreted cautiously as feedback was collected using an internal clinic survey rather than a psychometrically validated instrument.

### Limitations

Several areas of weakness should be acknowledged. This was a clinical sample so standardized timepoints for measurement were not established. Relatedly, some individuals were exposed to the treatment more frequently than others. While the clinician recommended certain exercises during at-home training, patients have the ability to work on any exercise in the program. Different patients may have engaged with different amounts of training in each cognitive domain. Because BrainHQ was utilized in a variable, self-directed manner at home, there was no standardized ‘dose’ of the intervention. While a dose–response analysis exploring the associations between total usage time, frequency of use, and changes in cognitive outcomes would be highly informative, the current study lacked the statistical power to conduct one. Only a subset of patients (*n* = 16) had both accessible usage data and at least one follow-up visit to assess the degree of change. This small sample size also restricted the complexity of the statistical modeling. However, it is worth noting that the primary objective cognitive metric, the ANAM Composite Score (ACS), is already a calculated z-score standardized against a normative sample based on age and sex, which partially mitigates any confounding effects of these demographic variables. Nonetheless, future prospective studies with larger cohorts are needed to isolate the specific therapeutic efficacy of cognitive training while controlling for factors such as age, education, and co-occurring symptoms.

Second, some individuals participating in the BFC were also participating in other rehabilitation services. While the intervention program in the BFC does target cognitive processing, it is possible the improvements noted here may be related to other interventions.

Third, without a matched control group, it is difficult to fully isolate true treatment effects from procedural learning or practice effects. Although the ANAM generates randomized stimuli, repeated exposure to the task demands can produce modest practice effects. However, as discussed previously, the magnitude of the improvement observed in our sample’s composite score exceeds the threshold expected from practice effects alone (44).

Fourth, the high rate of clinical attrition between the initial intake and subsequent follow-up visits limits the generalizability of these findings. While some causes of patients not returning were administrative or logistical (e.g., relocation, tele-health transitions), attrition bias remains a concern. It is possible that individuals who benefited the most, objectively or subjectively, from the intervention were more likely to return for care, while those who experienced less benefit, or those whose symptoms resolved completely, did not. Consequently, the follow-up data may represent a self-selected sub-population, and these observed clinical trajectories should not be generalized to all individuals reporting AHI.

Fifth, differences from normative comparison populations may differ if a control group could be better matched in terms of demographics and co-morbid factors (e.g., post-traumatic stress symptoms).

Sixth, patient satisfaction and program acceptability were measured using an internal clinic questionnaire without established psychometric properties. Consequently, these findings should be viewed as preliminary clinical feedback rather than validated measures of acceptability. Because the survey was administered directly by the treating clinic, responses may also be subject to social desirability bias, wherein patients might over-report their satisfaction or perceived cognitive benefits out of a desire to please the clinical staff.

Finally, this was not a homogeneous population. While all individuals had reported an AHI that led to their referral, there likely was variability in the etiology of those incidents.

### Future applications

Despite these limitations, the intervention described here appears well tolerated, acceptable to patients, and associated with a positive clinical trajectory in aspects of cognitive function in a complex patient population. These results provide some preliminary evidence supporting the DoD clinical practice recommendation that clinicians receiving referrals for patients with suspected AHI may consider adapting treatment protocols used for mTBI, such as implementing clinician-directed computer-based cognitive training in rehabilitation. These results also provide preliminary evidence for future prospective studies to address clinician-directed computer-based cognitive training as part of a regimen for cognitive rehabilitation in this population.

The combination of sub-clinical but statistically significant differences in objective cognitive performance on some components of the ANAM and high levels of reported functional impact suggest several future directions. First, future studies may investigate whether other objective measures besides the ANAM (e.g., more comprehensive measures with composite scores, measures that address modalities besides the visual modality) may capture information that better reflects functional concerns. Future studies may also investigate the relationship between patients’ expectations about their cognitive performance and their experience of subjective symptoms. Clinically, the profiles of self-reported concerns, lack of change in subjective measures, and patient reported importance of consultation with clinicians suggest that clinicians should also consider psychoeducation, for example to help patients connect cognitive gains to improvements in daily function and quality of life.

As there exists no literature describing specific cognitive rehabilitation interventions for this patient group (and only one other paper to our knowledge describing any rehabilitation intervention (46) in the population), we hope that this study advances the understanding and treatment of this important population.

## Data Availability

The data analyzed in this study is subject to the following licenses/restrictions: Summary/aggregate data and additional information on the methods and statistical analyses may be provided on request. However, individual data elements including self-report data and neuropsychological test data are not available due to DoW legal requirements. Requests to access these datasets should be directed to louis.m.french.civ@health.mil.
